# Practicing for life: landscape and priorities for simulation-based education in global children’s surgery

**DOI:** 10.1007/s00383-026-06607-z

**Published:** 2026-09-22

**Authors:** Yemi S. Lawrence, Chisom R. Udeigwe-Okeke, Nimanya A. Stella, Kara L. Faktor, Ramesh M. Nataraja, Caroline Q. Stephens, Doruk E. Ozgediz, Emmanuel A. Ameh

**Affiliations:** 1 University of California, San Francisco, CA USA; 2 National Hospital, Abuja, Nigeria; 3 National Referral Hospital, Kampala, Uganda; 4 Monash Children’¹s Hospital, Melbourne, Australia

**Keywords:** Simulation, Simulation-based education, Children’s surgery, Global surgery

## Abstract

**Purpose:**

Simulation-based education (SBE) is increasingly used in surgical training worldwide. With a shortage of specialized surgeons, SBE allows for skills acquisition in a safe environment, preserving patient safety and standardizing care. However, in global children’s surgery, knowledge about SBE interest, use, and feasibility is limited.

**Methods:**

A 17-item survey was developed to examine simulation in global children’s surgery. The survey was piloted (Nov 2024) and distributed (Dec 2024–Feb 2025) through the Global Initiative for Children Surgery (GICS), an organization with 1100+ members across 60 countries. Descriptive statistics and free responses were reported.

**Results:**

Of 85 respondents from 36 countries (approximately 7.7% response rate), over half used SBE in the past year, reaching about 1270 learners. The most common methods were low-cost models, mannequins, and bench trainers. Respondents identified several significant barriers to establishing SBE programs. Most (75%) simulation costs were under $300.

**Conclusion:**

Access to SBE resources remains a major barrier, highlighting the critical need for access to open-source education materials. The low cost suggests a need to evaluate cost-effectiveness. Despite a limited response rate and sampling from one organization, the use of low-cost simulation and interest in advanced technologies reflect a trend toward enhancing simulation in global children’s surgical education.

**Supplementary Information:**

The online version contains supplementary material available at https://doi.org/10.1007/s00383-026-06607-z.

## Introduction

An estimated 1.7 billion children lack access to safe, affordable, and timely surgical care with a higher representation in low and middle-income countries (LMICs) [1, 2]. A shortage of specialized surgeons, anesthesiologists and perioperative nurses constrain accessing surgical care in LMICs. Although there are approximately 1.1 million specialist surgeons worldwide, only 20% of them serve half of the global population living in LMICs [3]. As a result, task-shifting low-complexity, high-volume procedures, such as hernia repair and circumcision, to non-specialized surgeons and hospitalists has been evaluated as a means of expanding access to children’s surgical care [4], exposing a need for innovative surgical training programs to support the current workforce that cares for children’s surgical needs.

Simulation has become a growing part of surgical training programs and is recognized by the World Health Organization (WHO) as a priority for healthcare training [5]. Studies show an increasing use of simulation-based education (SBE) and improving clinical and patient outcomes across medical disciplines, including global surgery, with various technologies being used for training, evaluation of skills, and assessment of the quality of care provided [6–11]. SBE, as defined by international consensus, includes simulated patients, scenario-based simulations, mannequins, synthetic part-task trainers (PTT), animal PTT, cadavers PTT, bench trainers (i.e., laparoscopic trainers), extended reality (virtual/augmented/mixed reality), tele simulations, and computer/screen-based simulations [12]. Advanced technologies in SBE include 3D printing, artificial intelligence (AI), and extended realities.

Advancement in SBE has the potential to address the challenges of providing safe and accessible surgical care by building the capacity of the current workforce. Despite the growth in SBE and their advantages for preparing trainees, limited knowledge exists regarding the use and interest in simulation for global children’s surgery. Furthermore, limited studies address the feasibility and attitude towards children’s surgical simulation in LMICs setting [13]. Therefore, the aim of this study is to explore the landscape of SBE in global children’s surgery, including its current use, perceived effectiveness in achieving reported outcomes, priorities for future SBE training, and feasibility (technical, operational, economic) of implementing SBE methods through the Global Initiative for Children’s Surgery (GICS). Assessing existing simulation use and identifying priorities of educators in global children’s surgery is essential to build upon current initiatives.

## Materials and methods

### Participants

Participants were recruited through the GICS email list between December 2024 and February 2025. GICS was chosen for its extensive global reach of 1100 members from 60 low-, middle- and high- income countries (HICs). GICS members include all children’s specialties, general surgeons, anesthesia providers, pathologists, radiologists, nurses, and trainees, many of whom are actively engaged in global surgical education initiatives. As such, the sample reflected the broader global children’s surgery education community, representing individuals with expertise in children’s surgery and surgical training from diverse settings.

### Survey development

The survey was designed by a team of pediatric general surgeons, general surgery residents, medical student and surgeon educators from the USA, Uganda, Nigeria, and Australia. It encompassed 17 items, which included Likert scale questions, yes/no questions, and check-all-that-apply multiple choice questions (MCQs). Additionally, there was a short demographic section that gathered information on the participants’ country and, if applicable, the specialty-based GICS working group in which they are a member. Questions evaluated whether participants used simulations, and if not, whether they expressed interest in using simulations. For individuals utilizing SBE, questions asked about the modalities, procedures, teaching strategies, location, learner/educator characteristics, and intended learning outcomes. SBE modalities were limited to procedural based modalities to teach surgical skills for children’s surgery which included synthetic PTT defined as a partial model of the body made of synthetic materials to practice specific skills, animal PTT defined as a partial model of the body from an animal cadavers, cadavers PTT defined as a partial model of the body from human cadavers, bench trainers defined as laparoscopy trainers, extended reality (virtual/augmented/mixed reality), and computer/screen-based simulation. For individuals not using simulations but interested in SBE, priorities and barriers to implementing SBE were assessed. Perspectives on SBE in children’s surgery were evaluated for both groups.

Questions were informed by the TIDieR (Template for Intervention Description and Replication) guide and insights from experts in global surgical education, survey design, and simulation-based education research [14]. After a consensus on included items was reached among the survey team, the survey underwent review for applicability by an SBE expert (RMN) and the GICS publication committee. A pilot of the survey was conducted with four GICS members with expertise in surgical education from the USA and East Africa (Burundi, Uganda, Ethiopia) to gather feedback on the survey length, clarity of questions, and suggestions for improvement. This approach was in accordance with the Consensus-Based Checklist for Reporting of Survey Studies (CROSS) guidelines [15].

### Survey administration

The survey was distributed via email listserv hosted on Google Form, an online survey platform (Google, USA). Data was collected from December 17th, 2024, to February 28th, 2025, with a link to the survey sent three times during that time interval to ensure appropriate exposure and recruitment. The survey link collected email addresses to prevent multiple submissions from the same participant. The survey instrument is attached as Appendix 1. No incentives were offered to complete the survey.

### Data analysis

The study aimed to assess interest in and the current use of SBE to teach surgical skills for children’s surgery across learners at any stage of training within the GICS community. Simulations were therefore narrowed to procedural based training aimed at teaching surgical skills for children’s surgery. Responses were excluded if they included SBE methods involving only simulated patients, scenarios/role-play, or simulations focused on non-children’s surgical care. Duplicate responses (4) identified by the participant’s email address were removed, keeping the most recent response.

Survey responses were analyzed using descriptive statistics, presented as frequencies for baseline characteristics and percentages for categorical variables. Open-ended questions were reported descriptively. Data were analyzed using Microsoft Excel TM (Microsoft Corporation, Washington, USA) and R/R studio (R Core Team, Vienna, Austria; Posit Software, PBC, Boston, MA., USA). Data visualization using Datawrapper (Datawrapper GmbH, Berlin, Germany) and Microsoft Excel TM.

### Ethical considerations

Exempt status was obtained from the University of California, San Francisco (UCSF) Institutional Review Board (IRB) before the start of survey distribution. Consent was obtained from all participants when starting the survey. Survey responses collected were de-identified, password-protected, and accessible only to the study team to maintain confidentiality.

## Results

### Participants demographics

**Fig. 1:**
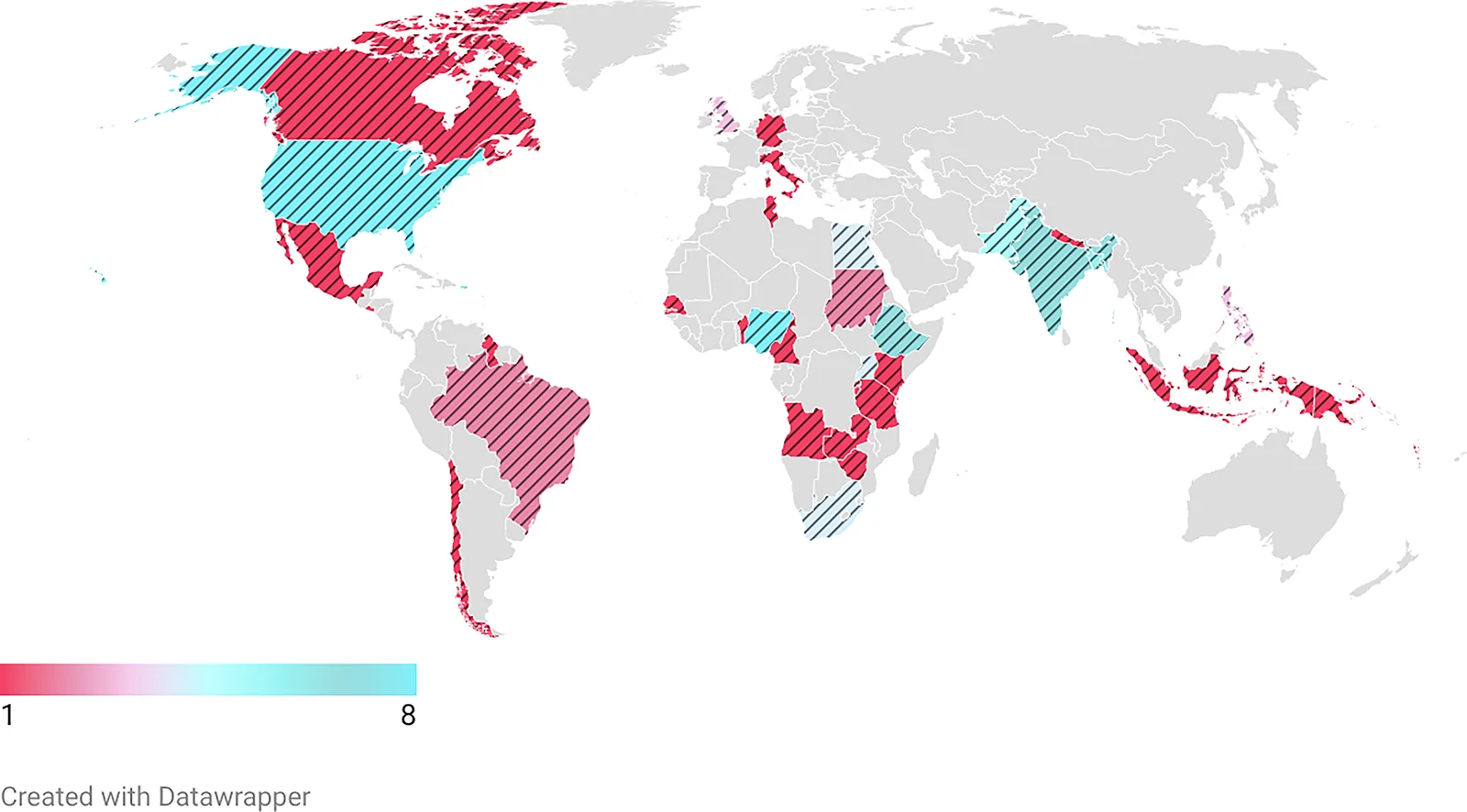
Global distribution of respondents

Eighty-five unique responses were collected across thirty-six countries, yielding an approximate 7.7% response rate. A total of 30 open-ended question responses were received. Countries with the highest number of responses included Nigeria (*n* = 8, 9%), India (*n* = 7, 8%), Ethiopia (*n* = 7, 8%), Bangladesh (*n* = 6, 7%), and the USA (*n* = 5, 6%) (Fig. 1). Half of the participants (43/85, 51%) were members of various GICS working groups and among the twelve different GICS working groups represented; most were involved in general surgery (52%), oncology (19%), and anesthesia (10%). Other groups included urology, trauma, orthopedic surgery, congenital anomalies, and nursing (Table 1). Most participants (76%) were consultants defined as senior physicians or surgeons that had completed all required training in their respective field (Table 1).

**Table 1:** Participants demographics and simulation use (*n* = 85)

Characteristics	Participants
Total countries	36
Regions, *n* (%)	
Africa	41 (48%)
Asia	23 (27%)
Europe	5 (6%)
Oceania	2 (2%)
Americas	15 (17%)
Simulation use, *n* (%)	
Yes	43 (51%)
No	42 (49%)
GICS Working Group (*N* = 43), n (%)	
General surgery	23 (52.38%)
Oncology	8 (19.05%)
Anesthesia	4 (9.52%)
Urology	2 (4.76%)
Congenital anomalies	2 (4.76%)
Trauma	1 (2.38%)
Orthopedics	1 (2.38%)
Other	2 (4.76%)
Level of training, *n* (%)	
Consultant	65 (76.4%)
Fellow	5 (6.0%)
Resident	1 (1.2%)
Nursing	1 (1.2%)
Non-clinical	1 (1.2%)
NA	12 (14%)

### Simulation priorities in global children’s surgery

**Fig. 2:**
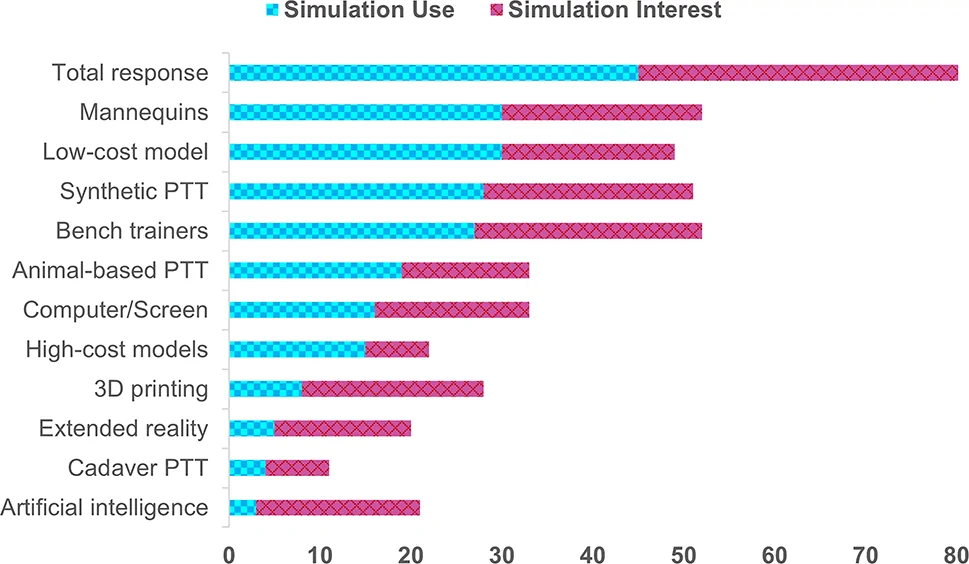
Simulation method use and interest

Forty-three respondents (51%) reported using SBE (Table 1). Of the respondents using SBE, the methods reported most frequently included low-cost models (*n* = 30, 66%), mannequins (*n* = 30, 66%), synthetic PTT (*n* = 28, 62%), and bench trainers defined as laparoscopic trainers (*n* = 27, 60%) (Fig. 2). 47% (47%) of these activities involved full operative procedures, with 32% focusing on complex interventions such as cleft lip repair, bowel resection, appendectomy, ostomy creation, inguinal hernia repair, endorectal pull-through, ventriculostomy, and vesicostomy creation.

Highly sought after SBE methods included bench trainers (*n* = 25, 61%), synthetic PTT (*n* = 23, 56%), mannequins (*n* = 22, 54%), 3D printing (*n* = 20, 49%), low-cost models (*n* = 19, 46%), and AI (*n* = 18, 44%) (Fig. 2). Highly sought after procedures included advanced sub-specialty procedures including tracheoesophageal fistula repair and posterior sagittal anorectoplasty (PSARP) (*n* = 30, 81%), endorectal pull through (*n* = 26, 72%), and inguinal hernia repair (*n* = 24, 66%). Participants also showed high interest in simulation of laparoscopy (*n* = 31, 76%). Open ended responses revealed interest in models for esophageal and biliary atresia (*n* = 4, 24%).

### Perceived effectiveness of simulations in achieving desired outcome

Survey results indicated that SBE techniques were perceived as very or extremely effective in achieving outcomes related to knowledge (*n* = 28, 64%), skills (*n* = 34, 75%), and behavioral change (*n* = 28, 64%). Perceptions of the effect of SBE on patient outcomes varied with 33% rating as not applicable (*n* = 15) and another 33% rating as very or extremely effective (*n* = 15) (Table 2). Knowledge outcomes were defined as knowledge acquisition evidenced by pre- and post-activity examinations. Skills outcome represented the development of specific skills, typically accompanied by pre/post practice with a simulation model, checklist assessment, comparisons of learners’ performance speed, assessment of the learning process, or evaluation of the end product. Perceived behavioral outcomes indicated changes in a learner’s confidence and comfort with model-focused skills. Patient outcomes were defined as the impact of simulation models on outcomes of actual patients.

**Table 2:** Simulation outcomes and perceived effectiveness (*n* = 45)

Simulationoutcomes *N* (%)	(5)Extremely effective	(4)Very effective	(3)Moderately effective	(2)Slightly effective	(1)Not effective	(0) NA
Knowledge	6 (14%)	19 (43%)	8 (18%)	2 (5%)	0 (0%)	9 (20%)
Skills	10 (22%)	20 (44%)	10 (22%)	2 (5%)	0 (0%)	3 (7%)
Behavior*	8 (18%)	17 (39%)	9 (20%)	2 (5%)	1 (2%)	7 (16%)
Patient outcomes**	5 (12%)	8 (19%)	13 (30%)	2 (5%)	1 (2%)	14 (33%)

*Changes in a learner’s confidence and comfort with modeled skills

**Impact of simulation training on outcomes of actual patients

### Barriers to establishing SBE

Significant barriers to establishing SBE included no dedicated educational spaces for simulation (*n* = 18, 46%), access to simulation teaching resources (*n* = 21, 55%), access to simulation technicians to maintain the models (*n* = 25, 63%), availability of distributors (*n* = 25, 63%), and insufficient funding (*n* = 37, 90%). Moderate constraints included limited educators (*n* = 17, 45%) and learner time (*n* = 16, 41%), experience with SBE techniques (*n* = 15, 39%) and a heavy clinical workload (*n* = 14, 37%). Learners’ interest (*n* = 16, 42%) in simulations was a minor constraint for establishing SBE. Access to the internet varied among participants, with 33% (*n* = 13) experiencing minor constraints and 31% (*n* = 12) facing moderate constraints.

### Feasibility of SBE in global children’s surgery

Residents (medical school graduate pursuing graduate medical education and training) (*n* = 37, 82%), fellows (physician undergoing advanced sub-specialized training) (*n* = 29, 64%) and children’s specialist surgeons (*n* = 32, 71%) were all reported as frequent participants in simulation sessions. The majority (*n* = 86, 80%) of the simulation initiatives were locally taught and occurred in a dedicated simulation center (*n* = 30, 68%). Other common locations included conferences (*n* = 17, 39%), hospital wards (*n* = 17, 39%), university classrooms (*n* = 14, 32%), and home/personal lodging (*n* = 11, 25%). In-person sessions (*n* = 39, 89%) were common, with in-person demonstrations (*n* = 35, 80%) and practice sessions (*n* = 32, 72%) serving as common teaching strategies. Simulation often coexisted with a didactic course (*n* = 19, 43%) while a portion (*n* = 12, 27%) occurred independently. Cost associated with simulations were defined as covering a single simulation unit, which included approximate production cost, materials, time, and equipment. The cost of simulations was predominantly under 300 USD with 50% reporting a range of 50–300 USD and 21% reporting cost < 50 USD. An overwhelming majority (*n* = 44, 90%) reported continued use of simulations for training as of 2024.

###  Perspectives of SBE in global children’s surgery

Globally, 96% (82/85) of participants expressed interest in using simulation for training in children’s surgical disease. Furthermore, 94% (79/84) indicated that access to content experts would enhance the simulation education they deliver. 94% (94%) agreed that simulation serves as a useful tool for building clinical knowledge (78/83) and procedural skills (79/84) necessary for treating children with surgical conditions globally. Additionally, 92% (77/84) expressed a desire to receive further training on SBE.

## Discussion

Most of the world’s population lacks access to surgical care, and even when access is achieved, the quality of care can be suboptimal [16]. Global children’s surgery access and outcomes remain inequitably distributed, with higher mortality from common children’s surgical conditions in low-resource settings [1, 16, 17]. SBE has the potential to play a pivotal role in developing technical skills, applying surgical knowledge, and acquiring competency in a safe, controlled environment. In the context of children’s surgery, where much of the global pediatric population resides in LMICs, it is essential to understand the priorities and barriers to establishing SBE within these settings. Findings from this study demonstrate an interest in SBE and a wide range of simulation modalities currently employed in children’s surgery worldwide. Prior literature has offered limited insight into the types of simulators used and SBE teaching strategies employed. Our study expands this knowledge, outlining simulation priorities, outcomes, feasibility, and associated barriers to establishing additional SBE initiatives in children’s surgery.

### Simulation priorities in global children’s surgery

Based on the survey findings, a wide range of SBE techniques are already being utilized, including mannequins, synthetic PTT, cadaver PTT, box trainers, and low-cost models. This is consistent with a systematic review of SBE in LMICs, which reported that mannequins and synthetic PTT were the most common modalities [6, 11]. However, the focus of reviews and simulation studies from LMIC have been centered in obstetric and neonatal medicine, with 75% coming from middle-income countries [6, 11, 13].

A review of simulations in children’s surgery showed a growing number of specialty-specific models, however, only 13% (5/38) of these models were available for purchase, and just 29% (11/38) could be replicated [18]. Although the number of models is increasing, the limited availability, high cost and poor reproducibility limits access to these tools in LMICs. This reinforces the need for low-resource, reusable and easily accessible models, a priority clearly reflected by our study’s respondents. Furthermore, fewer than 58% of documented SBE models met the validity threshold, highlighting the need not only for accessibility, but also models with demonstrated educational validity [18, 19].

There is also a significant interest in incorporating advanced technologies into SBE, such as computer simulations, 3D printing, extended reality, and artificial intelligence. Heo et al. (2024), while not specific to children’s surgery, found that web-based, application-based, virtual reality, and augmented reality were advanced technologies applied within surgical training in low-resource settings [7]. In fact, Puri et al. (2017) reported 203 studies using advanced technologies within LMICs, demonstrating growing use of these technologies within low-resource setting [6, 20]. Our findings were consistent with these prior studies showing that there are current use and interest in incorporating more advanced technologies within children’s surgery simulations. However, barriers such as cost, lack of technicians, and access to teaching resources still exist. Thus, collaboration will be key in accessing and adopting advanced technologies for simulation within global children’s surgery, enabling sharing of resources and models for widespread use.

Interest in simulating advanced subspecialty procedures is also evident, including among documented children’s surgery simulations in low-resource settings [21]. This includes full operative procedures, such as esophageal atresia repair, posterior sagittal anorectoplasty (PSARP) to extracorporeal membrane oxygenation (ECMO) [18, 21]. Our open-ended responses revealed a large interest in esophageal and biliary atresia models. Although one review noted that 79% of children’s simulations were focused on MIS surgery, all articles were from high- and middle-income countries [18].

###  Simulation outcomes and perceived effectiveness

The most frequently reported outcomes in this study included skills, followed by knowledge and behavior, with patient outcomes often not assessed, also seen in the review by Puri et al. focused on simulation within low-resource settings [6, 13]. Simulation in children’s surgery has served as both an assessment tool and a teaching resource, yet there are no reports of improvement in clinical outcomes [22]. Our findings highlight that there may be an underestimation of the simulation use in global children’s surgery. As new learning strategies emerge in SBE including the implementation of self-directed simulations [23] in children’s surgery [24, 25], there is an urgent need for more research focused on the impact of simulation on behavior and patient outcomes. It is important to demonstrate safety and quality of surgical care with simulations acquired skills, as these may pose barriers to future adoption and widespread simulation use [23]. Collaboration in organizations like GICS, with local experts leading initiatives, will be vital for implementing sustainable SBE and for enabling long-term follow-up on patient outcomes [26, 27].

### Barriers to SBE

Significant barriers to establishing SBE still exist, including access to teaching resources, highlighting the need for continual provision of open resource materials such as those offered by WHO Academy, TeachMeSurgery, and the Surgical Education Learners Forum (SELF) education platforms [28–30]. Additionally, challenges such as limited educator and learner time, inadequate space to conduct simulations, limited accessibility to simulation material distributors, and limited availability of qualified technicians for model maintenance remain inadequately addressed. Previous studies have described these barriers, emphasizing the lack of widespread availability despite increasing numbers of validated simulation models [18]. The same challenges apply to simulation facilities, which often have limited hours for trainees to access simulation models [20]. It is essential to prioritize accessibility as SBE initiatives are being pursued for children’s surgery. This includes both simulation facilities and training resource development. Studies have suggested the establishment of international technology repositories to promote accessibility and support global simulation efforts [7, 9, 31]. All these efforts require multinational collaboration. Irfanullah et al. (2023) and Bulamba et al. (2019) provide examples of how collaboration can lead to successful development of a new simulation center, implementation of training workshops, and support for low-cost simulation models [32, 33].

### Feasibility

In this study, the cost of most models was under 300 USD. This aligns with findings from other studies that illustrate the cost-effectiveness of surgical simulation [13, 21]. Rebello et al. 2025 indicated that the cost of children’s surgery models in low-resource settings ranged from 0.61 USD to 1219.18 USD [21]. However, it is important to emphasize reporting model cost in simulation research to help with this effort. One study of validated children’s surgery models revealed that only 37% (14/38) of models reported cost, while another study of procedural skill simulations in LMICs reported only 11% (29/262) provided cost data [13, 18]. Thus, reporting of model cost is needed to better understand cost-effectiveness of simulation models and work towards development of affordable, low-resource simulation models.

The majority (68%) of the simulations in this study take place in a dedicated simulation center in contrast to a prior study in LMICs reporting only 2% of simulations activities were conducted in dedicated simulation centers [6]. Additionally, many of the simulation sessions are led by local experts, who can provide context-specific and relevant teaching strategies for managing various surgical diseases. Puri et al. (2017) noted that 30% of LMIC instructors lack formal training in simulation [6]. Thus, open-access teaching resources may be an avenue to support local simulation instructors. Many studies do not comment on sustainability of SBE [13]. Thus, it is important to consider cultural context, affordability, and sustainability of simulation models as well as their durability and maintenance requirements [31]. Simulations should align with these criteria to be successfully implemented in global settings.

### Perspectives of SBE in global children’s surgery

Like in Robinson et al., most of our participants who utilize and express interest in simulations indicated a strong desire for additional SBE training [34]. The majority valued the contribution of content experts and saw simulations as a useful tool for knowledge and skills development in treating global children’s surgical diseases. Recent studies have focused on task-shifting initiatives that train general surgeons and hospitalists to perform low-complexity, high-volume surgical procedures, with early findings demonstrating comparable patient outcomes, although long-term follow-up, as well as evaluation in HIC settings, is still needed [4]. SBE appears to be a useful tool for training these providers, and our results suggest that simulations are likely to be well received in children’s surgery settings, especially when resources for additional SBE training and content-expert support are available.

### Limitation

This study has a few limitations. The findings from this study are based on data from a single organization, though it included participants from 36 countries. Moreover, the low response rate suggests that the sample may not represent the broader population of individuals interested in or using simulation for global children’s surgery in GICS. Factors that may have contributed to the low response rate include the length of the survey, timing of its distribution, and likely online survey fatigue. The effects of the low participation may limit the generalizability of the study findings and increase susceptibility to non-response bias. Furthermore, this study relied on self-reported data, making it vulnerable to recall bias. Despite these limitations, the sample remains representative of the target population of leaders in global children’s surgery and simulation education. Future studies should explore using incentives to enhance response rates.

## Conclusions

SBE is widely used in global surgery and shows an increasing presence in global children’s surgery. Our findings reveal a range of SBE methods, with a desire to enhance methods through advanced technologies. Overall, simulation priorities were geared toward subspecialty procedures. Access to teaching resources remains a significant barrier to establishing SBE, highlighting the need for open access education materials like the WHO Academy, TeachMeSurgery, and the Surgical Education Learners Forum (SELF). Despite the limitations of a single organization and response rate, the use of low-cost simulations and interest in additional teaching resources reflect a trend toward enhancing simulation in global children’s surgical education and highlights areas of further development in building capacity for children’s surgical care.

## Supplementary Information

Below is the link to the electronic supplementary material.

Supplementary Material 1

## Data Availability

The anonymized data collected by the survey are available as open data in the Dataverse repository: http://datadryad.org/share/LINK_NOT_FOR_PUBLICATION/Ldq0W7DyhCaYvIRHm_S6drkp7Glt4K14tDWrYEjPM_E.

## References

[1] Alkire BC, Raykar NP, Shrime MG et al (2015) Global access to surgical care: a modelling study. Lancet Glob Health 3:e316–323. 10.1016/S2214-109X(15)70115-4 25926087 PMC4820251

[2] Mullapudi B, Grabski D, Ameh E et al (2019) Estimates of number of children and adolescents without access to surgical care. Bull World Health Organ 97:254–258. 10.2471/BLT.18.216028 30940982 PMC6438256

[3] Holmer H, Lantz A, Kunjumen T et al (2015) Global distribution of surgeons, anaesthesiologists, and obstetricians. Lancet Glob Health 3:S9–S11. 10.1016/S2214-109X(14)70349-3 25926323

[4] Bognini MS, Oko CI, Kebede MA et al (2023) Assessing the impact of anaesthetic and surgical task-shifting globally: a systematic literature review. Health Policy Plan 38:960–994. 10.1093/heapol/czad059 37506040 PMC10506531

[5] WHO (2013) Transforming and scaling up health professionals’ education and training: World Health Organization guidelines 2013. World Health Organization, Geneva 26042324

[6] Puri L, Das J, Pai M et al (2017) Enhancing quality of medical care in low income and middle income countries through simulation-based initiatives: recommendations of the Simnovate Global Health Domain Group. BMJ Simul Technol Enhanc Learn 3:S15–S22. 10.1136/bmjstel-2016-000180

[7] Heo K, Cheng S, Joos E, Joharifard S (2024) Use of innovative technology in surgical training in resource-limited settings: a scoping review. J Surg Educ 81:243–256. 10.1016/j.jsurg.2023.11.004 38161100

[8] Elendu C, Amaechi DC, Okatta AU et al (2024) The impact of simulation-based training in medical education: a review. Med (Baltim) 103:e38813. 10.1097/MD.0000000000038813 PMC11224887 38968472

[9] Qayumi K, Badiei S, Zheng B et al (2014) Status of simulation in health care education: an international survey. Adv Med Educ Pract 10.2147/AMEP.S65451 PMC4257018 25489254

[10] Chou WK, Ullah N, Arjomandi Rad A et al (2022) Simulation training for obstetric emergencies in low- and lower-middle income countries: a systematic review. Eur J Obstet Gynecol Reprod Biol 276:74–81. 10.1016/j.ejogrb.2022.07.003 35820293

[11] Robinson SJA, Ritchie AMA, Pacilli M et al (2024) Simulation-based education of health workers in low- and middle-income countries: a systematic review. Glob Health Sci Pract 12:e2400187. 10.9745/GHSP-D-24-00187 39510603 PMC11666082

[12] Lioce L (2020) Healthcare simulation dictionary, second. Agency for healthcare research and quality

[13] Pollok F, Lund SB, Traynor MD et al (2023) Systematic review of procedural skill simulation in health care in low- and middle-income countries. Simul Healthc J Soc Simul Healthc. 10.1097/SIH.0000000000000737 37440427

[14] Hoffmann TC, Glasziou PP, Boutron I et al (2014) Better reporting of interventions: template for intervention description and replication (TIDieR) checklist and guide. BMJ 348:g1687. 10.1136/bmj.g1687 24609605

[15] Sharma A, Minh Duc NT, Luu Lam Thang T et al (2021) A consensus-based checklist for reporting of survey studies (CROSS). J Gen Intern Med 36:3179–3187. 10.1007/s11606-021-06737-1 33886027 PMC8481359

[16] Wright NJ, Smith ER, Bisquera A et al (2021) Paediatric surgical outcomes in sub-Saharan Africa: a multicentre, international, prospective cohort study. BMJ Glob Health 10.1136/bmjgh-2020-004406 PMC8413881 34475022

[17] Meara JG, Leather AJM, Hagander L et al (2015) Global Surgery 2030: evidence and solutions for achieving health, welfare, and economic development. Surgery 158:3–6. 10.1016/j.surg.2015.04.011 25987187

[18] Joosten M, De Blaauw I, Botden SM (2022) Validated simulation models in pediatric surgery: a review. J Pediatr Surg 57:876–886. 10.1016/j.jpedsurg.2022.06.015 35871858

[19] Patel EA, Aydın A, Desai A et al (2019) Current status of simulation-based training in pediatric surgery: a systematic review. J Pediatr Surg 54:1884–1893. 10.1016/j.jpedsurg.2018.11.019 30573294

[20] Milburn JA, Khera G, Hornby ST et al (2012) Introduction, availability and role of simulation in surgical education and training: Review of current evidence and recommendations from the Association of Surgeons in Training. Int J Surg 10:393–398. 10.1016/j.ijsu.2012.05.005 22609475

[21] Rebello L, Livergant R, Khanbadr P et al (2025) Simulation models for training in pediatric general, thoracic, plastic, and urologic surgery in low-resource settings: a scoping review. J Pediatr Surg 60:162183. 10.1016/j.jpedsurg.2025.162183 39890497

[22] Yokoyama S, Mizunuma K, Kurashima Y et al (2019) Evaluation methods and impact of simulation-based training in pediatric surgery: a systematic review. Pediatr Surg Int 35:1085–1094. 10.1007/s00383-019-04539-5 31396735

[23] Fariyike OA, Yao J, Baqri M et al (2024) See one, teach yourself one, do one: Barriers and opportunities in self-administered training and assessment for global surgical education. Surg Open Sci 22:74–78. 10.1016/j.sopen.2024.11.001 39649290 PMC11621498

[24] Colostomy in Newborns In: appropedia. https://www.appropedia.org/Colostomy_in_Newborns#Overview

[25] Safe Paediatric Male Circumcision Simulator In: appropedia. https://www.appropedia.org/Safe_Paediatric_Male_Circumcision_Simulator

[26] GICS (2022) Global Initiative for Children’s Surgery. In: Glob Initiat Child Surg https://www.globalchildrenssurgery.org/

[27] Ameh EA (2023) Realigning global health realities towards children’s surgery: progress and possibilities. J Pediatr Surg 58:1039–1047. 10.1016/j.jpedsurg.2023.02.006 36907771

[28] WHO Academy In: WHO.int. https://www.who.int/about/who-academy

[29] Teach Me Surgery https://teachmesurgery.com/

[30] Surgical Education Learners Forum https://www.appropedia.org/SELF

[31] Bolton WS, Aruparayil N, Quyn A et al (2019) Disseminating technology in global surgery. Br J Surg 106:e34–e43. 10.1002/bjs.11036 30620068

[32] Irfanullah EA, Chandra A, Solaiman RH et al (2023) Simulation training in a lower middle-income country: supporting a new center and developing low-cost models for critical skill acquisition. Cureus. 10.7759/cureus.40950 37503495 PMC10368800

[33] Bulamba F, Sendagire C, Kintu A et al (2019) Feasibility of simulation-based medical education in a low-income country: challenges and solutions from a 3-year pilot program in Uganda. Simul Healthc J Soc Simul Healthc 14:113–120. 10.1097/SIH.0000000000000345 30601468

[34] Robinson SJA, McLeod E, Nestel D et al (2024) Simulation-based education in the Pacific Islands: educational experience, access, and perspectives of healthcare workers. ANZ J Surg 94:2021–2029. 10.1111/ans.19188 39205429

