# Supplementary material for: Practicing for life: landscape and priorities for simulation-based education in global children’s surgery
Source: Pediatr Surg Int. 2026 Sep 22;42(1):418. doi: 10.1007/s00383-026-06607-z (PMC13597628; doi:10.1007/s00383-026-06607-z)
Supplement: Supplementary file 1 [file 383_2026_6607_MOESM1_ESM.pdf]

Practicing for Life: Landscape and Priorities for Simulation-Based Education in Global Children's Surgery

Pediatric Surgery International

Yemi S. Lawrence*, B.S University of California, San Francisco, San Francisco, CA

yemi.lawrence@ucsf.edu. 916-256-9350 ORCID: 0000-0002-8718-3174

Chisom R. Udeigwe-Okeke*, MBBS, FWACS, National Hospital, Abuja, Nigeria, FWACS,

Nimanya A. Stella*, MBChB, Mulago National Referral Hospital, Kampala, Uganda

Kara Faktor, MD, MSc, University of California, San Francisco, San Francisco, CA

Ramesh M. Nataraja, MBBS, MSurgicalEd, FRCSEd(Paed), Monash Children's Hospital, Melbourne, Australia

Caroline Stephens, MD, MPH, University of California, San Francisco, San Francisco, CA

Doruk E. Ozgediz, MD, FACS, University of California, San Francisco, San Francisco, CA

Emmanuel A. Ameh, MBBS, PhD, FACS(Hon), National Hospital, Abuja, Nigeria, on behalf of the Global Initiative for Children's Surgery

*These authors contributed equally to this work

GICS Simulation Survey 2024

Thank you for your participation in the Global Initiative of Children Surgery (GICS) Simulation Survey 2024. Our goal through this survey is to build a comprehensive understanding of the **interest in** simulations, the **current practices** around simulation-based education (SBE), and the use of advanced technologies in SBE within the GICS consortium. SBE, as defined by international consensus, includes standardized patients, scenario-based simulations, mannequins, synthetic part-task trainers (PTT), animal-based PTT, cadavers PTT, bench trainers (ie laparoscopic trainers), extended reality (virtual/augmented/mixed reality), tele simulations, and computer/screen-based simulations. Advanced technologies in SBE include 3D printing, artificial intelligence (AI), and extended realities. We hope to better understand the interest in simulation use in children's surgery education within our organization and the feasibility of simulation use in global pediatric surgical work and capacity building. Thank you for completing the survey and contributing to this initiative.

SURVEY QUESTIONS

1. Country ________________
2. **Are you a member of a GICS working group? Please select YN**
    a. If Yes, please click the name of the GICS working group you are a member of:
        i. Anesthesia
        ii. Cardiac surgery
        iii. Congenital Anomalies
        iv. Critical care
        v. Dental / Oral surgery
        vi. Family support
        vii. General surgery
        viii. Neurosurgery
        ix. Nursing
        x. Oncology
        xi. Ophthalmology
        xii. Orthopedic
        xiii. Otolaryngology
        xiv. Pediatric General surgery
        xv. Plastic Surgery
        xvi. Radiology
        xvii. Trauma
        xviii. Urology
        xix. Other working group ________________
    b. No, continue survey
3. Are you (or your team) currently using or developing simulations? Please select YN
    a. If Yes, continue with the survey
    b. If No, only answer Q4-6, and Q17
4. What type of simulations would you (and your team) be *interested* in? Check all that apply:
    a. Mannequins (defined as a life-like model of the body)

    b. Synthetic part task trainers (PTT) (defined as a partial model of the body made of synthetic materials to practice specific skills. For example, a model of the arm to practice blood draw)
    c. Animal-based part task trainers (PTT)
    d. Cadaver part task trainers (PTT)
    e. Bench trainers (defined as laparoscopic trainers)
    f. Computer/screen-based simulator (defined as PC/computer-simulated environment, screened-based simulation)
    g. 3D printing (defined as the use of 3D printing of all or some aspect of the model)
    h. Extended reality (defined as virtual reality, augmented reality, and mixed reality)
    i. Artificial intelligence (defined as AI-generated or use of AI within the simulation)
    j. Low-cost model (Model may be made with simple supplies like gloves, boxes, paper, tissues, etc)
    k. High-cost models (Model may be made with complex supplies like silicone, rubber, metal, wood, etc)
    l. Other types of simulation modality _________________
    m. Not interested in simulation-based education
5. **Please rank your level of interest** from 0 (not interested) to 4 (very interested) for the following procedure/skills you would like to have simulated models for:
    a. Suturing
    b. Wound care
    c. Pelvic packing
    d. Airway management
    e. Inferior Alveolar Nerve (IAN) block
    f. Central Line placement
    g. Ventriculostomy
    h. Ear tube placement
    i. Chest tube placement
    j. Peritoneal Drain placement
    k. Appendectomy
    l. Ostomy creation
    m. Vesicostomy creation
    n. Bowel resection/ anastomosis
    o. Inguinal hernia repair
    p. Endorectal pull through
    q. Osteomyelitis debridement
    r. External fixation
    s. Advanced sub-specialty procedure (ie TEF, Posterior sagittal anorectoplasty (PSARP)
    t. Laparoscopy simulator
    u. Other procedure/skills_________________
6. **Please rank the following** constraints from 0 (not a constraint) to 4 (a significant constraint) for starting simulation-based education:
    a. Space (no dedicated sim center or space to set up simulations)
    b. Financial

c. Limited educator time
d. Limited learner time
e. Lack of learner interest in using simulators
f. Lack of hospital/administration support
g. Heavy clinical workload
h. Experience with simulation-based education techniques
i. Access to instructors/content experts
j. Access to simulation-based education teaching resources
k. Access to simulation technicians and upkeep of models
l. Access to reliable internet
m. Access to simulators/models (ie access to the model/material distributor)
n. Other challenges/comments:________________

7. What type of simulations have you (and your team) used or are currently using? Check all that apply:
   a. Mannequins (defined as a life-like model of the body)
   b. Synthetic part task trainers (PTT) (defined as a partial model of the body made of synthetic materials to practice specific skills. For ex, a model of the arm to practice blood draw)
   c. Animal-based part task trainers (PTT)
   d. Cadaver part task trainers (PTT)
   e. Bench trainers (defined as laparoscopic trainers)
   f. Computer/screen-based simulator (defined as PC/computer-simulated environment, screened-based simulation)
   g. 3D printing (defined as the use of 3D printing of all or some aspect of the model)
   h. Extended reality (defined as virtual reality, augmented reality, and mixed reality)
   i. Artificial intelligence (defined as AI-generated or use of AI within the simulation)
   j. Low-cost model (model may be made with simple supplies like gloves, boxes, paper, tissues, etc)
   k. High-cost models (model may be made with complex supplies like silicone, rubber, metal, wood, etc)
   l. Other types of simulation modality ________________
8. What procedure or skill are the simulation models being used for? Check all that apply:
   a. Suturing
   b. Wound care
   c. Pelvic packing
   d. Airway management
   e. Inferior Alveolar Nerve (IAN) block
   f. Central Line placement
   g. Ventriculostomy
   h. Ear tube placement
   i. Chest tube placement
   j. Peritoneal Drain placement
   k. Appendectomy
   l. Ostomy creation

m. Vesicostomy creation
n. Bowel resection /anastomosis
o. Inguinal hernia repair
p. Endorectal pull through
q. Osteomyelitis debridement
r. External fixation
s. Advanced sub-specialty procedure (ie TEF, Posterior sagittal anorectoplasty (PSAR)
t. Laparoscopy simulator
u. Other procedure/skills________________

9. Which learners are taking part in simulation-based education (SBE)? Check all that apply:
   a. Medical student (in the process of getting M.D/MBBS)
   b. Medical Doctor in training (resident). Please indicate year________________
   c. Medical Doctor in fellowship (fellow, subspeciality)
   d. Pediatric Surgeon
   e. General Surgeon
   f. Other surgical specialist
   g. Anesthesiologist
   h. Medical officer
   i. Nonsurgical specialist
   j. Other healthcare providers (nursing, etc)
   k. Other learners __________________
10. What teaching and learning strategies are used with the simulation model? Check all that apply:
    a. Face-to-face / in-person sessions
    b. Zoom / online sessions
    c. In-person demonstrations of the simulation model
    d. Online tutorials and video demonstrations of the simulation model
    e. In-person practice with simulation model
    f. Independent practice with simulation model (ie take home simulation model)
    g. Simulation coexisted with a didactic course
    h. Simulation existed on its own without an associated didactic course
    i. Other teaching/learning strategies________________
11. Where is the simulation-based education taking place?
    a. Dedicated simulation center
    b. Affiliated hospital ward
    c. Community health centre
    d. University or college classroom
    e. Conferences
    f. Home/ personal lodging
    g. Other location ____________________
12. Who is running the simulation sessions and teaching learners? Check all that apply:
    a. Local nonsurgical physician
    b. Local General Surgeon
    c. Local Pediatric surgeon
    d. Local surgical specialist (ie other surgical specialist)

e. Local Anesthesiologist
f. Local Medical Doctor in training (resident)
g. Local Medical Doctor in fellowship (fellow)
h. Other healthcare providers (nurses, midwife, etc)
i. Allied Health and other healthcare workers (ie Community health workers)
j. Visiting Physician (nonlocal physician)
k. NGO group. If Yes, Country of origin ____________________
l. Other (please specify) ____________________

13. Within the **last year**, how many learners have attended your group's simulation-based education (SBE) sessions?
    a. 0-10
    b. 11-20
    c. 21-30
    d. 31-40
    e. 41-50
    f. >50. If greater than 50, include an approximate estimate of the number of total participants that have attended the simulation session led by your group
14. Indicate the intended outcomes of the simulation and rank the effectiveness in achieving those outcomes from not effective to very effective. Check all that apply:
    a. Knowledge (to gain knowledge. A pre/post-multiple-choice question exam may have accompanied simulation)
    b. Skills (to develop specific skills. Simulations may have been accompanied by pre/post practice with a simulation model, checklist assessment, comparing how fast the learners were, assessing learning process, or assessing learner end product)
    c. Behaviors (to change participants' views. Simulators may have been accompanied by a survey assessing the impact on learners' confidence and comfortability doing the specific simulation model-focused skills)
    d. Patient outcome (Simulation models were assessed by their impact on patient outcomes)
    e. Other outcome______________________________
15. Are the simulators being used as of October 2024? YN
16. What is a single simulation unit's approximate production cost (ie, materials, time, equipment)?
    a. <50 USD
    b. 51-150 USD
    c. 151-300 USD
    d. > 300 USD. If greater than 300 USD, include an approximate estimate of the total production cost of the simulation models ___________USD
17. **The following set of questions** assesses perspectives around simulations

	Strongly Agree	Somewhat Agree	Neither Agree / Disagree	Somewhat Disagree	Strongly Disagree
I am *interested* in using simulation for training in children's surgical disease					

Simulation *is generally* overseen by content experts. Access to such content experts would enhance the simulation education that I deliver.					
Simulation is a *useful* tool for building clinical knowledge to treat children with surgical conditions in a global setting					
Simulation is a *useful* tool for building clinical procedural skills to treat children with surgical conditions in a global setting					
I would like additional educational training to enhance my educational practice using SBE					

If possible, provide information on where the materials and simulators can be accessed (for example, published paper PMID, online appendix, website URL)____

Would you be open to being contacted further about the simulations you are doing and/or simulations you are doing in the future? YN

That concludes the end of the survey. Thank you for completing the simulation survey! We appreciate your time and effort in doing so.

Stay in touch to hear more information about the simulation survey results!

Contact information

First and Last Name_______________________
Title/Role ____
Email address_______________________________
